# Millisecond photonic sintering of iron oxide doped alumina ceramic coatings

**DOI:** 10.1038/s41598-021-82896-9

**Published:** 2021-02-11

**Authors:** Evgeniia Gilshtein, Stefan Pfeiffer, Marta D. Rossell, Jordi Sastre, Lovro Gorjan, Rolf Erni, Ayodhya N. Tiwari, Thomas Graule, Yaroslav E. Romanyuk

**Affiliations:** 1 Laboratory for Thin Films and Photovoltaics, Empa - Swiss Federal Laboratories for Materials Science and Technology, Überlandstrasse 129, 8600 Dübendorf, Switzerland; 2 Laboratory for High Performance Ceramics, Empa - Swiss Federal Laboratories for Materials Science and Technology, Überlandstrasse 129, 8600 Dübendorf, Switzerland; 3 Electron Microscopy Center, Empa - Swiss Federal Laboratories for Materials Science and Technology, Überlandstrasse 129, 8600 Dübendorf, Switzerland

**Keywords:** Characterization and analytical techniques, Design, synthesis and processing, Synthesis and processing

## Abstract

The sintering of alumina (Al_2_O_3_) traditionally occurs at high temperatures (up to ca. 1700 °C) and in significantly long times (up to several hours), which are required for the consolidation of the material by diffusion processes. Here we investigate the photonic sintering of alumina particles using millisecond flash lamp irradiation with extreme heating rates up to 10^8^ K/min. The limitation of the low visible light absorption of alumina is resolved by adding colored α-Fe_2_O_3_ nanoparticles, which initiated the grain growth during sintering. After the millisecond-long light pulses from a xenon flash lamp, a bimodal mixture of α-Al_2_O_3_ precursor particles was sintered and iron segregation at the grain boundaries was observed. The proposed photonic sintering approach based on doping with colored centers may be extended to other refractory ceramics with low absorption in the visible light range once appropriate high-absorbing dopants are identified.

## Introduction

Fast sintering of alumina is already practiced for bulk alumina parts. During fast firing, samples are introduced for short times (up to several minutes) in preheated furnaces^1–3^ to rapidly expose samples to the temperatures at which densification mechanisms dominate^4,5^. This method allows sintering of only fine-grain sizes^5^ since a high sintering activity and enhanced stress relaxation by creep are needed^2^. A reduction in the necessary sintering temperature and activation energy compared to those needed for conventional furnaces was reported for fast sintering with a microwave plasma^6,7^ and self-propagation high-temperature synthesis (SHS)^8^. In addition, spark plasma sintering^9,10^ and flash sintering^11^ showed the possibility of producing dense alumina within 60 s by fast resistive heating. Another method called ultrafast high-temperature sintering (UHS), where the required sintering temperature can be reached in less than 30 s by positioning the ceramic precursor pellet in-between two Joule-heating carbon strips was recently demonstrated^12^. Laser sintering of bulk Al_2_O_3_ with different additives (ZrO_2_, WO_3_, and Cr_2_O_3_) was possible by a continuous-wave CO_2_ laser with irradiation times below 30 s but a partial melting was observed^13,14^.

When it comes to thin films or coatings of alumina, one can either sinter a precursor layer deposited by spin coating, dip coating, and electrophoretic deposition followed by annealing in a furnace^15^ or grow alumina layer from the vapor phase onto a heated substrate, e.g. by plasma spraying^16^. Thermal spraying and laser cladding are based on the occurrence of melting (temperatures above 2050 °C for alumina) with subsequent material recrystallization^15,17^, which restricts the choice of suitable substrates and can result in an uncontrolled grain structure^17–19^. Ultrathin alumina films with the thickness of several nanometers can be grown by chemical vapor deposition^20^ or atomic layer deposition^21^ at substrate temperatures as low as 100–200 °C, although such coatings are usually amorphous.

Here, we propose photonic sintering (also called flash lamp annealing (FLA)) to sinter alumina films on substrates with a melting point much lower than that of alumina. FLA employs ultrashort (0.1 to 10 ms) pulses of white light from a broadband xenon flash lamp to induce rapid transient heating of illuminated surfaces. Because heat is generated primarily within the surface layer, this method allows fast annealing of thin films even on temperature-sensitive substrates. In this study, soda-lime glass (SLG) has been selected as a model low-temperature substrate with a softening point at 720 °C that is significantly lower than the melting point of alumina. Similar sintering process was observed also on polycrystalline alumina and silicon wafer substrates. FLA is commonly used in printed and flexible electronics for annealing opaque materials, such as printable Ag, Cu, or carbon-based inks^22,23^. Only a few oxide materials with either a lower melting temperature (e.g., CuO^24^) or a much smaller bandgap (e.g., ZnO^25^) than alumina have already been processed by FLA. The main obstacle in the photonic processing of alumina (bandgap of 7–8 eV) is its pristine white color and hence a high optical reflectance, which limits the absorption of the visible light. To circumvent this obstacle, we employ a bimodal mixture of micron- and submicron-sized α-Al_2_O_3_ precursor particles together with reddish-brown-colored α-Fe_2_O_3_ nanoparticles that boost the optical absorptivity of the precursor layer.

## Results

**Figure 1:**
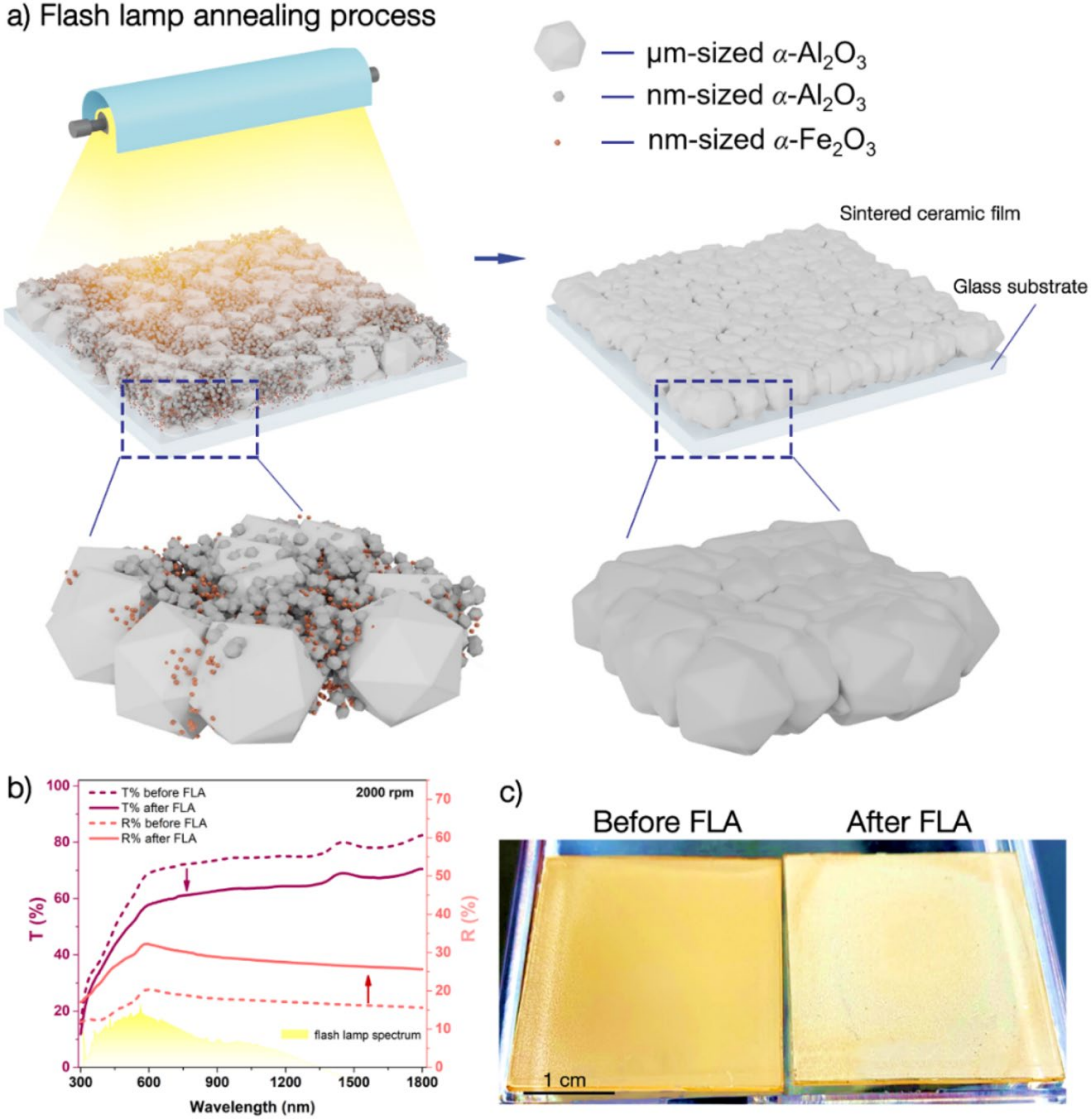
Flash lamp annealing (photonic sintering) process of alumina thin films. (**a**) Schematic illustration of the photonic sintering process of the precursor film, comprised of µm-sized α-Al_2_O_3_, nm-sized α-Al_2_O_3_ particles, and nm-sized α-Fe_2_O_3_ nanoparticles. (**b**) Optical transmittance and reflectance spectra of ceramic films before and after FLA. (**c**) Photographs of the ceramic films before and after FLA.

Figure 1a describes the process of photonic sintering of the alumina precursor layer with the α-Fe_2_O_3_ nanoparticles. The density and uniformity of the prepared coating layer were major factors that affected the multidirectional consolidation during the sintering of α-Al_2_O_3_-based films. To achieve a thin and uniform coating layer, we utilized a bimodal mixture of micron-sized (µm-sized) α-Al_2_O_3_, submicron (nm-sized) α-Al_2_O_3_, and doping of 1 wt% Fe_2_O_3_ nanoparticles, which is described in the “Materials and methods” section. The slurry solution was spin coated to obtain the ceramic layer on a glass substrate and dried at 120 °C on a hot plate for 20 min (Supplementary Fig. 1). The minimum layer thickness was limited by µm-sized α-Al_2_O_3_ particles with an average size of 3 μm. The absolute layer density was evaluated by referring to the volume percentages of the slurry ingredients and was calculated to be 71.5% of the theoretical layer density. Photonic sintering of the ceramic layers was performed in an air atmosphere with a xenon flash lamp. Since the emission spectrum of the xenon lamp (Fig. 1b) had a broad peak in the visible region (400–800 nm), absorption obtained by the Fe_2_O_3_ dopant was considered to be effective for the subsequent FLA. Five light pulses of 2.5 ms with the total exposure energy density of 125 J/cm^2^ were applied to the ceramic layer until visible whitening of the ceramic layer occurred. The processed ceramic film revealed a sintered grain morphology, which was analyzed in this study and is schematically shown in Fig. 1a.

As a result of the sintering process, the reflectance of the layer drastically increased (Fig. 1b), and the color of the layer changed from an intense orange to a light yellow (Fig. 1c). The layer color whitening, which suggests that sintering occurred, was homogeneous over the whole area; however, the edges appeared darker due to the substrate edge effect, which was ascribed to the perturbations during the spin coating process.

**Figure 2:**
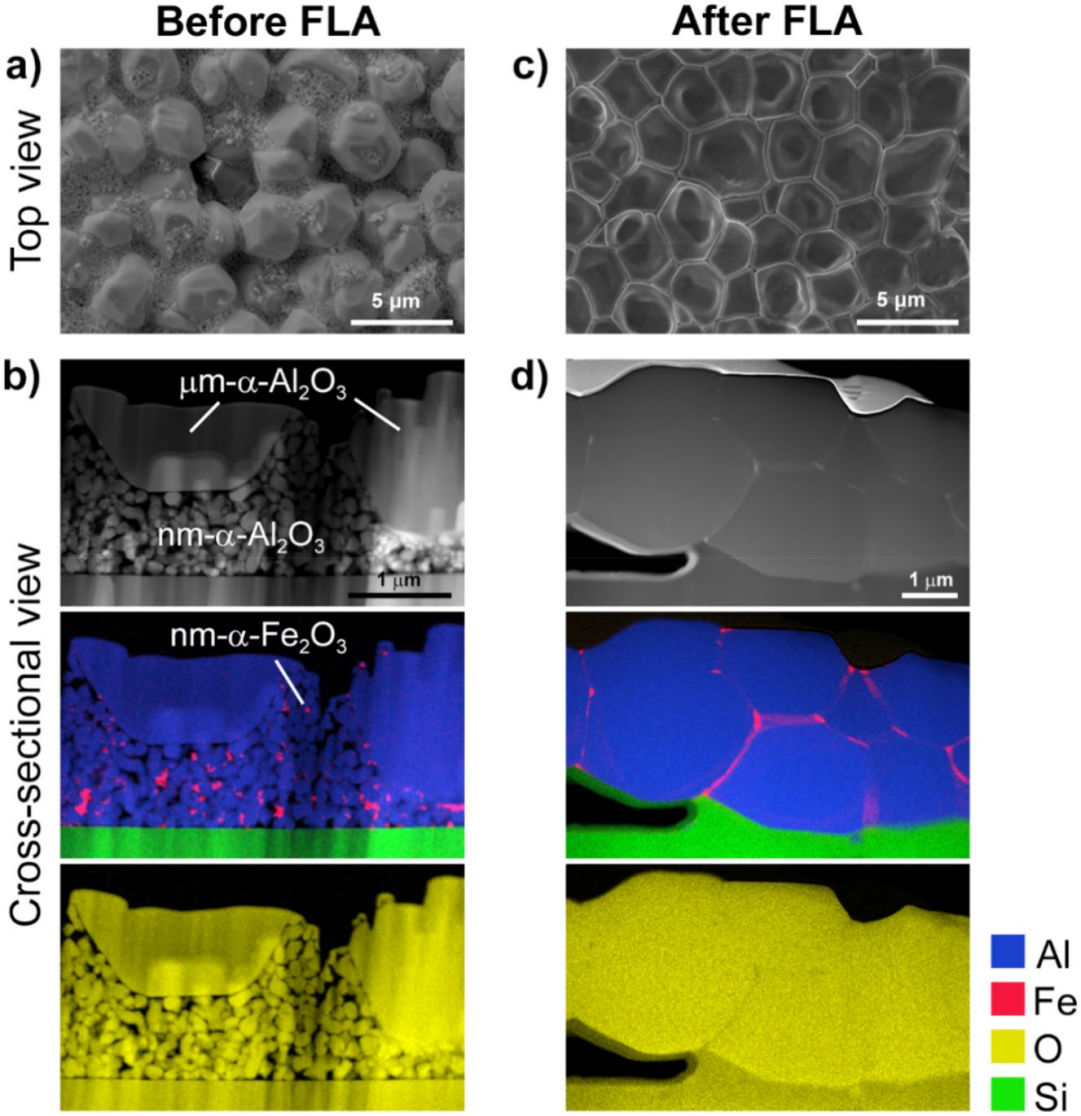
Compositional analysis of the ceramic film before and after FLA. The ceramic film before FLA: (**a**) Top-view SEM and (**b**) cross-sectional STEM images. The EDX elemental maps of the FIB cross-section show the film composition and distribution of Fe (α-Fe_2_O_3_ nanoparticles). Ceramic film after FLA: (**c**) top-view SEM and (**d**) cross-sectional STEM images. The EDX elemental maps of the FIB cross-section show the composition of the sintered film as well as the Fe localization at the grain boundaries.

Scanning electron microscopy (SEM) images (Fig. 2a–d) illustrate the morphology changes of the film, which occurred as a result of photonic sintering. The initial mixture of µm- and nm-sized α-Al_2_O_3_ particles with α-Fe_2_O_3_ dopant nanoparticles (Fig. 2a,b) was now visible in the form of sintered grains (Fig. 2c,d) with an average in-plane size of 2.35 µm and lateral size of 1.60 µm, as determined by the intercept method. The average grain dimensions were smaller than those of the initial micron-sized α-Al_2_O_3_ particles, which was mainly due to fast diffusion processes induced by the high activation energy of the flash lamp source. This result shows that diffusion processes occurred not only for the highly active nanoparticles but also for the micron-sized alumina particles. The results in this work are in contrast with those for other rapid sintering methods, where it was stated that only fine (submicron) grain sizes can be sintered quickly^5^ due to the high sintering activity and the enhanced stress relaxation by creep^2^. However, if short sintering time and fast solid-phase sintering reaction considered, then smaller grain sizes must be produced compared to the grains produced by conventional furnace–sintering^12^. We performed energy-dispersive X-ray spectroscopy (EDS) mapping analysis in a scanning transmission electron microscope (STEM) for a lamella obtained from the as-prepared spin-coated layer and sintered layer prepared by using a focused Ga^+^ ion beam (FIB) instrument. Al and O elements were dominant since these are the matrix elements in the bimodal α-Al_2_O_3_ mixture, whereas Fe in the form of α-Fe_2_O_3_ nanoparticles or nm-sized agglomerates were distributed homogeneously throughout the layer thickness before FLA (Fig. 2b). The α-Fe_2_O_3_ nanoparticles were assumed to initiate grain growth due to effective light absorption causing the local melting after FLA. Fe was present at the grain boundaries when sintering occurred (Fig. 2d). In contrast to the previously reported results for conventionally sintered Fe_2_O_3_-doped Al_2_O_3_^26^, iron atoms did not enter the alumina lattice here. The samples from the above-mentioned references were rapidly exposed to temperatures where densification mechanisms, such as from the aluminum lattice, grain boundaries, and volume diffusion, were dominant^4,5^. The amount of iron oxide dopant is extremely low in our study to form hercynite or other recrystallization products, in the case liquid form formation is assumed. At low temperatures, the activation enthalpy for these diffusion mechanisms is higher than for surface diffusion and evaporation/condensation mechanisms, which lead only to coarsening. The fact that iron was segregated at the grain boundaries could be explained by the larger ionic radius of Fe^3+^ than that of Al^3+^, which made its diffusion through the lattice difficult during the fast photonic sintering process. Based on that we assume that the rapid heating of the film through radiation and conduction of the solid-state reaction and ultra-fast sintering are presented.

The application of photonic sintering to layers composed of only α-Al_2_O_3_ without the incorporation of α-Fe_2_O_3_ was also investigated. The experiments demonstrated no effect of photonic sintering on the α-Al_2_O_3_ layers and the absence of morphological changes (Supplementary Fig. 2). This means that the effective application of the proposed method is only possible in the presence of a light-absorbing dopant, such as α-Fe_2_O_3_. We confirmed the feasibility of this method for a system containing only nm-sized α-Al_2_O_3_ particles with α-Fe_2_O_3_ doping (Supplementary Fig. 3). With this mono-modal alumina nanoparticle-based system, sintering also occurred, but the sintering activity of this system was too high, and thus, the accumulation of the thermal stresses resulted in very pronounced cracks and layer delamination. Thus, a bimodal mixture of alumina particles is preferred for uniform layer sintering.

In addition, photonic sintering experiments were carried out for the same α-Al_2_O_3_ bimodal mixture but with different amounts of the α-Fe_2_O_3_ dopant (Supplementary Fig. 4). The concentration of α-Fe_2_O_3_ nanoparticles for the material system with 0.5 wt% was not sufficient to initiate grain growth after exposure to the flash lamp source. Material systems with 2 wt% demonstrated a matrix that was oversaturated with α-Fe_2_O_3_ nanoparticles, which led to extremely harsh interactions with the flash lamp and cracking and delamination of the ceramic layer. We thus demonstrated that 1 wt% doping of α-Fe_2_O_3_ nanoparticles is the optimal amount for the α-Al_2_O_3_ layer sintering.

**Figure 3:**
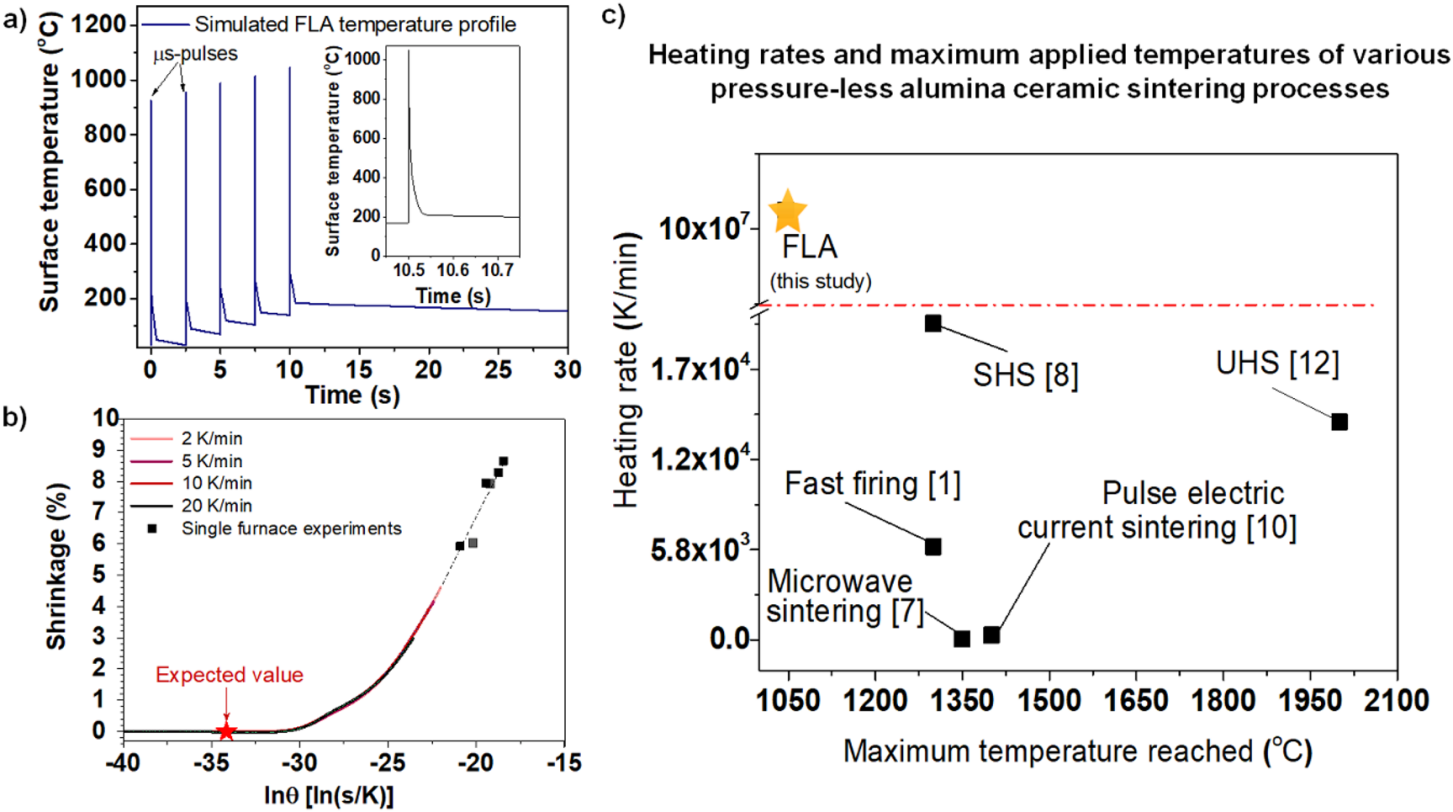
Characterization of the sintering process. (**a**) Simulated temperature profile obtained using SimPulse PulseForge 1300 built-in software (https://www.novacentrix.com/products/simpulse) image shows enlarged single peak of FLA microsecond process. (**b**)—inset Shrinkage of compressed Al_2_O_3_-Fe_2_O_3_ powders concerning activation energy, time, and temperature term ϴ in accordance with the Master Sintering Curve concept. (**c**) Heating rate for reported sintered alumina ceramics versus maximum applied temperature during the sintering process.

The application of the pulse parameters mentioned previously onto the optimized ceramic material system (a bimodal alumina mixture with 1 wt% α-Fe_2_O_3_) with the given optical and thermal properties was simulated by using SimPulse software. The maximum annealing temperature of 1045 °C during the FLA process was estimated for the 3 µm alumina film on a 1 mm SLG substrate as the input simulation structure. Based on the simulated temperature profile (Fig. 3a), a master sintering curve (Fig. 3b) was developed to compare the predicted shrinkage behavior of the grains (in accordance with conventional sintering) with the relative shrinkage of the layer after photonic sintering. The determined apparent activation energy of the powder-based system was 680 kJ/mol, which is slightly higher than the activation energy of 520 kJ/mol evaluated by Brosnan et al.^7^. However, in contrast to our study with a high amount of micron-sized α-Al_2_O_3_ grains, only nm-sized alumina was used. The predicted shrinkage value for photonic sintering of the ceramic layer according to the master sintering curve was 0% based on the given thermal history, the bimodal distribution of alumina, and a maximum temperature of 1045 °C with the sintering time of only 2.5 ms for each of the five pulses. For comparison, the layer shrinkage value extracted from the SEM images is 5.38%, which corresponds to a − 21.4 lnϴ value on the master sintering curve (fitted dashed line from single furnace experiments). These results confirm that due to the enhanced sintering rates of the rapid heating process, a decreased activation energy, and sintering temperature are required^6–8,11^. In the conventional furnace sintering process (used for the evaluation of the master sintering curve), the shrinkage started at 875 °C with a heating rate of 2 K/min and at 950 °C with a heating rate of 20 K/min, which are temperatures below the maximal reached pulse temperature of 1045 °C. Brosnan et al.^7^ referred to the lower activation energy in microwave sintering than that for conventional sintering as the reason that the diffusion is enhanced during the fast process. Furthermore, Ji et al.^8^ ascribed the accelerated sintering rate during fast sintering to the increased diffusion coefficients of non-equilibrium grain boundaries, which are formed due to an insufficient time for relaxation. The ultrafast sintering method presented in this work enables total sintering times of a few seconds and heating rates of 10^8^ K/min, which is more than four orders of magnitude faster than those reported previously for fast ceramic sintering processes (Fig. 3c). Due to the microsecond single pulse duration (inset Fig. 3a), it is possible to achieve temperatures required for the sintering process within microseconds and return to mild temperature conditions on the surface of the coating.

**Figure 4:**
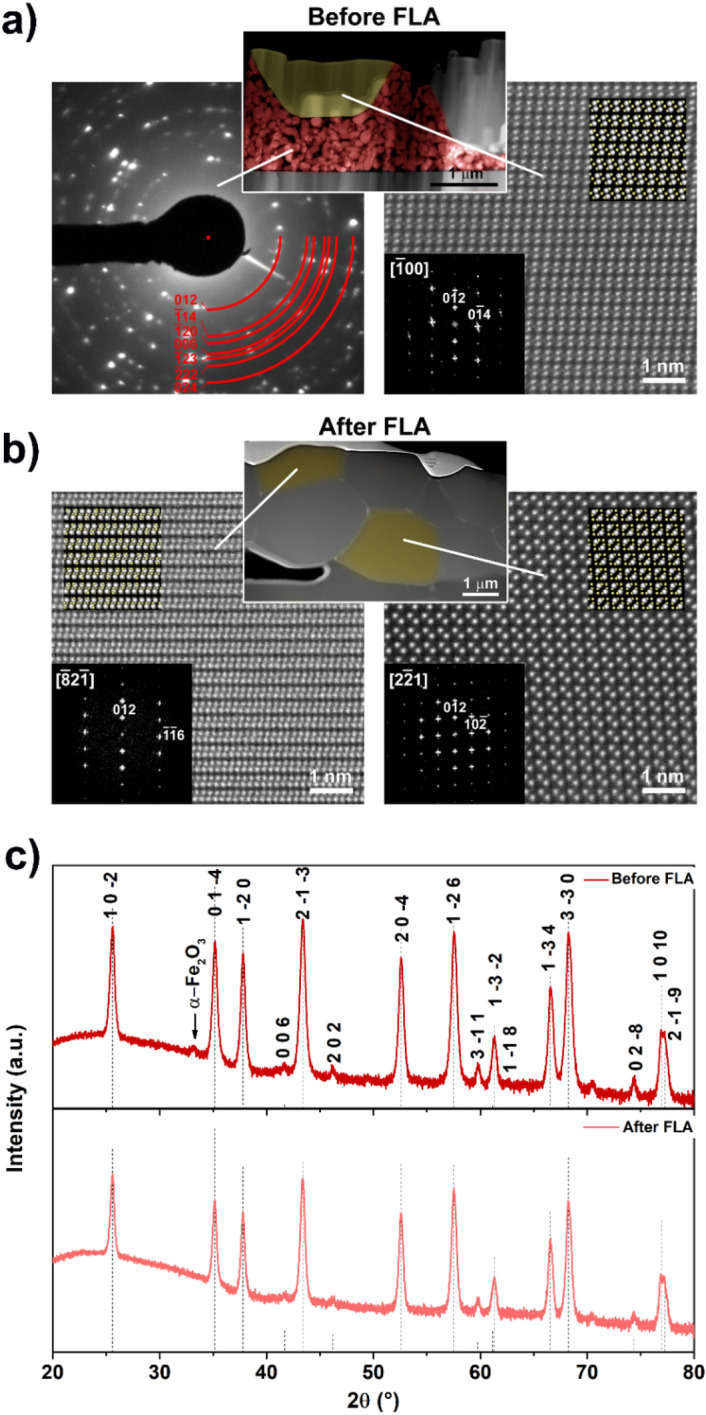
Structural characterization of the ceramic film before and after the FLA process. (**a**) Ceramic film before FLA: electron diffraction pattern and high-resolution HAADF-STEM image acquired from the nm-sized α-Al_2_O_3_ and α-Fel_2_O_3_ particles (indicated in red in the low-magnification HAADF-STEM image) and the µm-sized α-Al_2_O_3_ particle (shown in yellow), respectively. (**b**) Ceramic film after FLA: HAADF-STEM images acquired from the µm-sized α-Al_2_O_3_ particles highlighted in yellow. The electron diffraction pattern and the Fourier transforms of the HAADF-STEM images are all indexed using the α-phase of Al_2_O_3_. Models of the α-phase of Al_2_O_3_ along the corresponding zone axis are shown as insets; the Al and O atoms are shown in white and yellow, respectively. (**c**) XRD patterns of the ceramic films before and after FLA.

The structural properties of the sintered ceramic film were further examined by high-resolution high-angle annular dark-field scanning transmission electron microscopy (HAADF–STEM) analysis (Fig. 4a,b). Similar to the growth of the initial bimodal mixture of the µm-sized (shown in yellow in the low-magnification HAADF-STEM image of Fig. 4a) and the nm-sized α-Al_2_O_3_ particles (Fig. 4a—indicated in red), the sintered grains crystallized in the trigonal space group (Fig. 4b). The electron diffraction patterns and the Fourier transform of the HAADF-STEM images are all indexed to the α-phase of Al_2_O_3_ for the layers analyzed before and after sintering. Models of the α-phase of Al_2_O_3_ along the corresponding zone axis are shown as insets in the Fourier transform images.

This was additionally confirmed by X-ray diffraction (XRD) analysis of the layers in grazing incidence diffraction mode before and after FLA (Fig. 4c). Both patterns match well with the diffraction pattern of trigonal α-Al_2_O_3_ (reference pattern ICSD 52648). The peak at approximately 34° for the unprocessed layer corresponds to α-Fe_2_O_3_ and is not present in the diffraction image collected from the layer processed with FLA, which explains melting of the light-absorbing iron oxide agent. The remaining high-temperature-stable Al_2_O_3_ α-phase confirms that no melting most likely occurred during the processing of the layers. This is in contrast with the behavior of other methods, such as laser cladding^27^ and thermal spraying^28^, which are based on melting with subsequent recrystallization, where rapid cooling can maintain γ-Al_2_O_3_.

**Figure 5:**
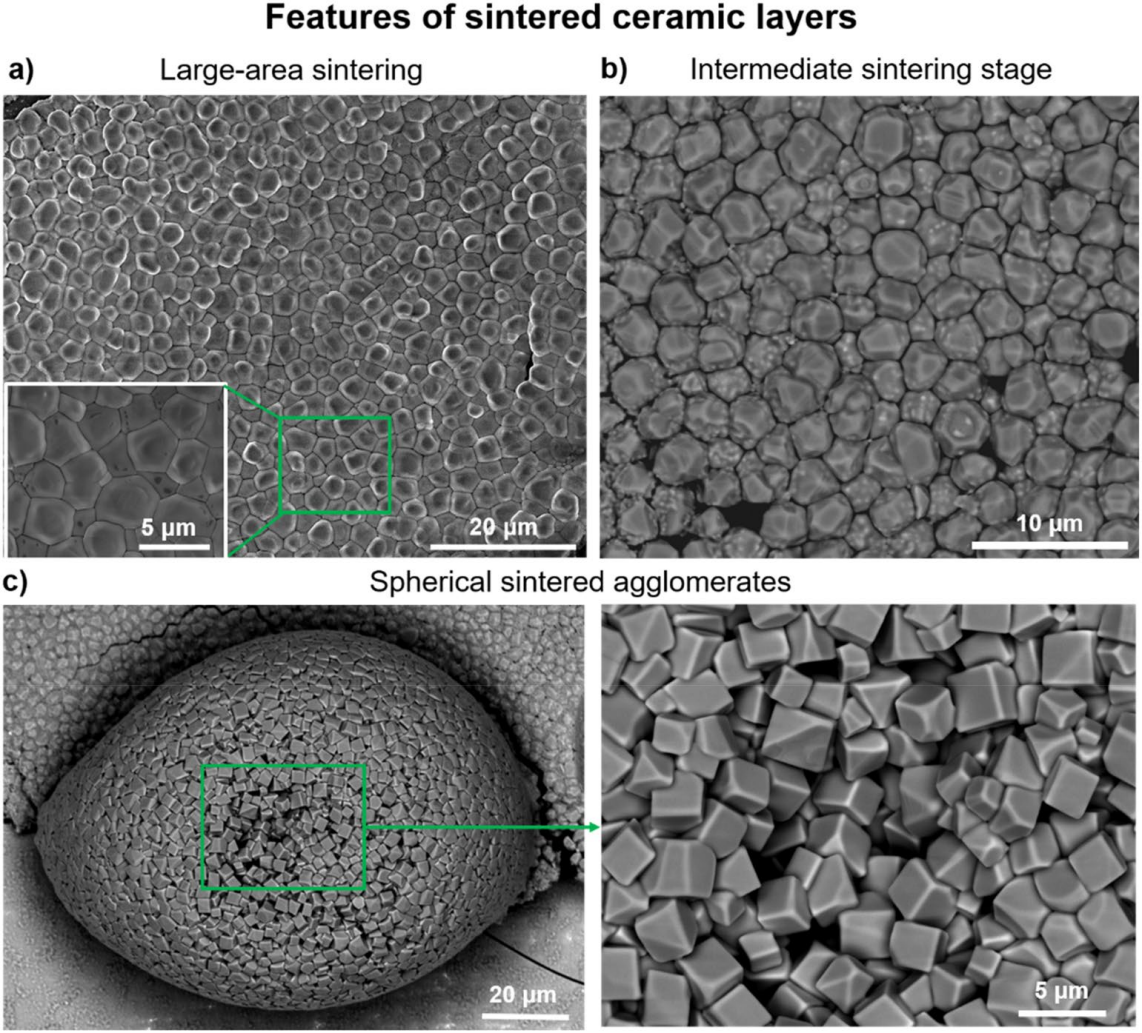
Morphological features and irregularities observed on FLA processed ceramic films. (**a**) SEM top view image of sintered film area (120 µm × 120 µm), inset reveals sintered grains of the layer. (**b**) SEM image of the area appearing in the intermediate sintering state (initiation of the grain growth). (**c**) SEM images of sintered sphere at different magnifications (spheres are located at the border of the sintered film).

Sintered areas of ceramic coatings can be obtained with the proposed photonic sintering method (Fig. 5a) with a crack-free area size of up to 200 µm × 200 µm depending on the initial coating uniformity (Supplementary Fig. 5). For some samples, parts were detected where the pulse energy was enough to initiate particle sintering and grain growth but not enough for the total consumption of the nanograins by the micron-sized grains (Fig. 5b). Assuming a three-dimensional shrinkage of 5.38% in each layer direction and a layer density of 71.5% before photonic sintering, the calculated porosity of the aluminum oxide layer should be 13.2% after photonic sintering. For the large area SEM images, 5 areas were analyzed and the in-plane porosity was found to be in the range of 6.2–8.3%. However, it is hard to evaluate the experimentally obtained porosity within the layer thickness, since during the FIB manipulation the cross-section is polished and therefore pores cannot be tracked anymore.

Interestingly, SEM analysis shows that all of the processed ceramic coatings exhibited large sintered spheres (Fig. 5c) at the borders of the processed layer. These spheres were composed of truncated cubic-like crystals. FIB cuts across the spheres revealed that they were hollow with wall thicknesses in the 3–10 µm range (Supplementary Fig. 6a, b). The EDX elemental maps of the FIB cross-section show the composition of one of the sintered spheres and the Fe localization at the grain boundaries, which is identical to the results obtained for the sintered layers. The HAADF-STEM images acquired from different grains and corresponding Fourier transforms (Supplementary Fig. 6c) were all indexed to the Al_2_O_3_ α-phase. One possible factor, which could lead to the formation of the spheres, is the balling effect due to the partial melting of the ceramic layer, which is known from laser processing to be the most favorable shape during melting of alumina ceramics^29^. However, the fact that these spheres were hollow and composed of only α-Al_2_O_3_ grains is contradictory to the assumption of balling, since rapid cooling from the melt most likely leads to the γ-Al_2_O_3_ formation, as described before. Another explanation of sphere formation could be the delamination and warping of some coating parts accompanied by ultrafast sintering. Due to the ultrafast sintering process, the system has no chance to compensate for the residual stresses, which are also caused by the shrinkage of the ceramic layer and by the difference in the thermal expansions of the glass substrate and the ceramic layer. Furthermore, the thickness of the sphere walls is in the range of the deposited layer thickness, which supports this assumption.

## Conclusions

We present how the addition of colored iron oxide particles allows rapid photonic sintering of alumina films & coatings. Thin alumina ceramic films (up to 3 µm) can be sintered on a glass substrate with a softening point of 720 °C that is substantially lower than the melting point of alumina—2072 °C. The sintering process occurred in the precursor layers comprising a bimodal α-Al_2_O_3_ particle mixture due to the effective doping by 1 wt% Fe_2_O_3_ nanoparticles, which resulted in extremely fast heating rates of up to 10^8^ K/min. The high-temperature-stable Al_2_O_3_ α-phase remained unchanged and iron was found to segregate at the grain boundaries. The fast heating and cooling, however, increase the chances of film cracking, and therefore substrates with similar thermal expansion are preferred.

The proposed approach of adding coloring agents into a transparent matrix can be extended to other refractory materials with low absorption in the visible light range, such as zirconia, titania, and alumina–zirconia composites, once appropriate high-absorbing dopants are identified. The application field could be broadened to the rapid 3D printing of ceramics by employing technologies such as ink-jet printing or screen printing.

## Materials and methods

### Particle dispersion

Nano α-Al_2_O_3_ Taimicron TM-DAR (Taimei Chemicals Co. LTD), micron α-Al_2_O_3_ AA3 (Sumitomo, Chemical Co. LTD) and nano-α/γ-Fe_2_O_3_ L2715D (BASF SE) were purchased as ceramic raw materials. Ammonium citrate dibasic p.a. 98% (Sigma Aldrich Corp.) was used as a surfactant to achieve a homogeneous dispersion of all particles in Nanopure water. For the nano-α/γ-Fe_2_O_3_ L2715D a BET average particle size was calculated from the absolute density and the corresponding specific surface area (SSA) assuming monomodal spherical particles according to the Sauter mean diameter^29^. BET (Brunauer–Emmett–Teller) measurements (SA 3100 Surface Area Analyzer, Beckman Coulter, Germany) provided the specific surface area (SSA) of the particles. Prior to the measurements, the raw powders were degassed with synthetic air for two hours at 180 °C to remove adsorbed water from the surfaces (SA-PREP Surface Area Outgasser, Beckman Coulter, Germany). The absolute densities of all powders were measured by helium pycnometry (AccuPyc II 1340, Micromeritics, USA).

Ammonium citrate dibasic was previously proven as a suitable dispersant for the used ceramic particles (Fe_2_O_3_ and Al_2_O_3_) in water, since it creates a negative surface charge on the particle surfaces by exchange of hydroxyl groups with carboxylate ions^30,31^. Saturation amount evaluation of dispersant on the particle surfaces by zeta-potential measurements as well as consecutive volume-based differential particle size distributions measured by dynamic light scattering (DLS) and laser diffraction (LD) can be found in Pfeiffer et al.^30^. Table S1 summarizes the measured absolute density, SSA and calculated BET average particle size and Table S2 shows d_10_, d_50_ and d_90_ measured by DLS and LD. A thin and uniform coating layer was achieved by utilizing a bimodal mixture of micron-sized (µm-sized) α-Al_2_O_3_, submicron (nm-sized) α-Al_2_O_3_, and doping of 1 wt% Fe_2_O_3_ nanoparticles, which was previously used for the selective laser melting (SLM) method^29,30^. Both corundum (α-Al_2_O_3_) and hematite (Fe_2_O_3_) belong to the same space group ${\text{R}}\overline3{\text{c}}$ with lattice parameters of a = 4.760 Å and c = 12.993 Å and a = 5.039 Å and c = 13.740 Å, respectively, and can form solid solutions. To achieve a high powder bed density, a bimodal distribution of alumina with a ratio of 72.6 vol% of micron-sized AA3 and 27.4 vol% of nano-sized Taimicron TM-DAR was chosen in accordance to Ref.^30,32^. Additionally, 1 wt% of Fe_2_O_3_ in respect to the entire inorganic content was added to the dispersion. The total solid load in the dispersion was 50 vol%. The absolute density of the spin coated film from slurry was evaluated by dividing the weight difference of the SLG substrate before and after spin coating with the measured volume of the layer.

### Flash lamp annealing and temperature simulations

FLA of the alumina films doped with 1 wt% of Fe_2_O_3_ was performed in the air atmosphere with a photonic curing system (NovaCentrix PulseForge 1300). For the FLA processes, samples were positioned 10 mm away from a Xe arc lamp with the face of the ceramic film directed toward the incident light. One pulse had a 2500 μs envelope comprising five 400 μs micro pulses with a 100 μs break after each micro pulse. Repeating the FLA treatment lead to the enlarging of the sintered film area. Therefore, consequent FLA pulses, using 850 V lamp voltage with five pulses repetition (with a total output exposure energy density of 125 J/cm^2^) were applied. These pulse parameters and repetition numbers were extracted after a series of experiments until ceramic exhibited unchanged whitish color.

In order to estimate the temperatures reached on the surface of the ceramic coating the SimPulse tool was used, which couples a transient 1-D heat conduction model to temperature dependent thermal and material properties^33^. For the simulations the FLA pulses were treated as a volumetric source heat flux for the materials stack: 1 mm thick SLG and 3 µm thick alumina, taking into account the correction of the coating absorptivity (~ 33%) due to the presence of Fe_2_O_3_ nanoparticles, which are considered to be homogeneously distributed within the thickness of the coating layer. Thermal conductivity of 36 W/mK, specific heat of 762 J/kg∙K, melting temperature of 2072 °C and heat fusion of 111 kJ/mol were used as input parameters for the alumina coating layer in the simulations.

### Master sintering curve

The use of the master sintering curve (MSC) model^34,35^ offers the possibility to predict shrinkage behavior from different firing profiles. There is no restriction to only one of the sintering stages, which means that the sinter ability of a compact can be measured over wide density range. If the requirements of microstructure evolution dependency from the density and only one dominant diffusion mechanism are met, the MSC pictures the expected densification/shrinkage from parts with the same staring powder and green body characteristics. Furthermore, it offers the possibility to control the shrinkage behavior by adjusting the temperature profile. In this model the function of density or linear shrinkage Φ(ρ), which include microstructural and material properties, can be described as a function of temperature T and time t. The parameters related to the microstructure evolution and the temperature terms are on the opposite side of the equation. It is a measure of accumulative effect of high temperature exposure on the shrinkage. According to the MSC different firing profiles can lead to the same value of temperature and time dependent function ϴ(t,T(t)) and accordingly to the same densification. This theta function is based on Arrhenius exponential function. By utilization of the apparent activation energy for sintering Q and the gas constant R this function can be calculated as:

$$\Phi (\rho )=\theta (t,T\left(t\right))= \underset{0}{\overset{t}{\int }}\frac{1}{T} {e}^{\left(- \frac{Q}{R T}\right)} dt$$

In order to display the master sintering curve, various steps were carried out. First, the dispersed powders were spray dried by the aid of the binder PEG 35000 (Sigma Aldrich Corp.). Afterwards the spray-dried granules were molded into 7.8 mm × 10 mm cylinders by uniaxial pressing with 520 MPa, followed by heating with 2 K/min to 900 °C in air inside a Profitherm PY 12 H furnace (PYROTEC Brennofenbau GmbH) to remove the organic content (2 wt% binder and 0.25 wt% dispersant). Subsequently, the shrinkage behavior was measured by thermo-mechanical analysis with TMA 402 F3 Hyperian (Netzsch GmbH) in a temperature range from 700 to 1500 °C in air atmosphere for the different heating rates of 2, 5, 10 and 20 K/min. The initial densities after pressing were between 71.6 and 72.1% and thus comparable to the evaluated density of 71.5% for the spin coated layer prior to photonic sintering.

By determination of an appropriate apparent activation energy, the four different shrinkage in respect to ln ϴ curves can be converged together. This single curve is called the master sintering curve, which is unique for a given powder and green-body density and characterizes the sintering behavior regardless of the used thermal history^34,35^. To describe the shrinkage capability of the bimodal powder compact, experiments in air with temperatures up to 1750 °C in the high temperature furnace LHT 04/17 (Nabertherm GmbH) were conducted in order to evaluate single points with smaller log ϴ on the master sintering curve.

### Characterization methods

Thickness and layer morphology were studied by SEM measurements (FEI Quanta 650 SEM). The average grain size of the SEM micrograph in Fig. 2c,d was determined by the interception method. Randomly positioned line segments were drawn on the micrograph and the number of intersects at a grain boundary was counted. The grain size was then calculated as ratio of intercepts to total line length. Relative shrinkage was calculated as fraction of the difference between to average layer thickness before and after flash lamp annealing over average layer thickness after spin coating.

Electron transparent cross-sectioned samples for transmission electron microscopy were prepared by means of a FEI Helios NanoLab 600i focused ion beam (FIB) operated at accelerating voltage of 30 and 5 kV. High-angle annular dark-field scanning transmission electron microscopy (HAADF-STEM) and energy dispersive X-ray (EDX) spectroscopy maps were carried out on a probe aberration-corrected Titan Themis microscope operated at 300 kV and equipped with ChemiSTEM technology. Elemental maps were calculated from the EDX spectrum image using the O-K_α_, Al-K_α_, Si-K_α_ and Fe-K_α_ lines. Electron diffraction (ED) was performed using a JEOL 2200FS TEM/STEM microscope operated at 200 keV.

Transmittance was measured with a UV–Vis spectrometer Shimadzu UV-3600 from 250 to 1500 nm taking air as the reference (baseline).

XRD patterns of the thin films were recorded in a grazing incidence (GI) mode with an incident angle ω = 2° over the range 2θ = 20°–80° (in steps of 0.01° and a scan rate of 3 s/step) using a Bruker D8 Discover diffractometer with Cu K_α_ radiation.

## Supplementary Information

Supplementary Information.
