# Supplementary material for: Millisecond photonic sintering of iron oxide doped alumina ceramic coatings
Source: Sci Rep. 2021 Feb 11;11:3536. doi: 10.1038/s41598-021-82896-9 (PMC7878514; doi:10.1038/s41598-021-82896-9)
Supplement: Supplementary file 1 — Supplementary Information. [file 41598_2021_82896_MOESM1_ESM.docx]

**Supplementary Information**

**Millisecond photonic sintering of iron oxide doped alumina ceramic coatings**

Evgeniia Gilshtein^1+,*^, Stefan Pfeiffer^2+,*^, Marta D. Rossell^3^, Jordi Sastre^1^, Lovro Gorjan^2^, Rolf Erni^3^, Ayodhya N. Tiwari^1^, Thomas Graule^2^, Yaroslav E. Romanyuk^1,*^

^1^ Laboratory for Thin Films and Photovoltaics, Empa – Swiss Federal Laboratories for Materi-als Science and Technology, Überlandstrasse 129, 8600 Dübendorf, Switzerland

^2^ Laboratory for High Performance Ceramics, Empa – Swiss Federal Laboratories for Materi-als Science and Technology, Überlandstrasse 129, 8600 Dübendorf, Switzerland

^3^ Electron Microscopy Center, Empa – Swiss Federal Laboratories for Materials Science and Technology, Überlandstrasse 129, 8600 Dübendorf, Switzerland

^+^ Evgeniia Gilshtein and Stefan Pfeiffer contribute equally to this paper.

**Keywords:** Flash lamp annealing, Intense pulsed light, ceramic coating, alumina, iron oxide

**Table S1.** Absolute density and specific surface area (SSA) of raw powders measured by helium pycnomerty and BET measurements, respectively, and calculated BET average particle sizes for all powders.

|  | absolute density [g/cm^3^] | SSA [m^2^/g] | BET average particle size [nm] |
| --- | --- | --- | --- |
| Al_2_O_3_ AA3 | 4.01 | 0.40 | 3756 |
| Al2O3 Taimicron TM-Dar | 3.95 | 11.80 | 129 |
| Fe_2_O_3_ L2715D | 4.38 | 69.73 | 20 |

**Table S2**. d_10_, d_50_ and d_90_ of volume based particle size distributions of dispersed powders in water determined by dynamic light scattering (DLS) and laser diffraction (LD).

| Powder | Al_2_O_3_ AA3 | Al_2_O_3_ Taimicron TM-DAR | | Fe_2_O_3_ L2715D | |
| --- | --- | --- | --- | --- | --- |
| Measurement method | LD | LD | DLS | LD | DLS |
| d_10_ | 2.2 μm | 109 nm | 133 nm | 51 nm | 37 nm |
| d_50_ | 3.0 μm | 148 nm | 201 nm | 79 nm | 53 nm |
| d_90_ | 4.4 μm | 212 nm | 295 nm | 110 nm | 82 nm |


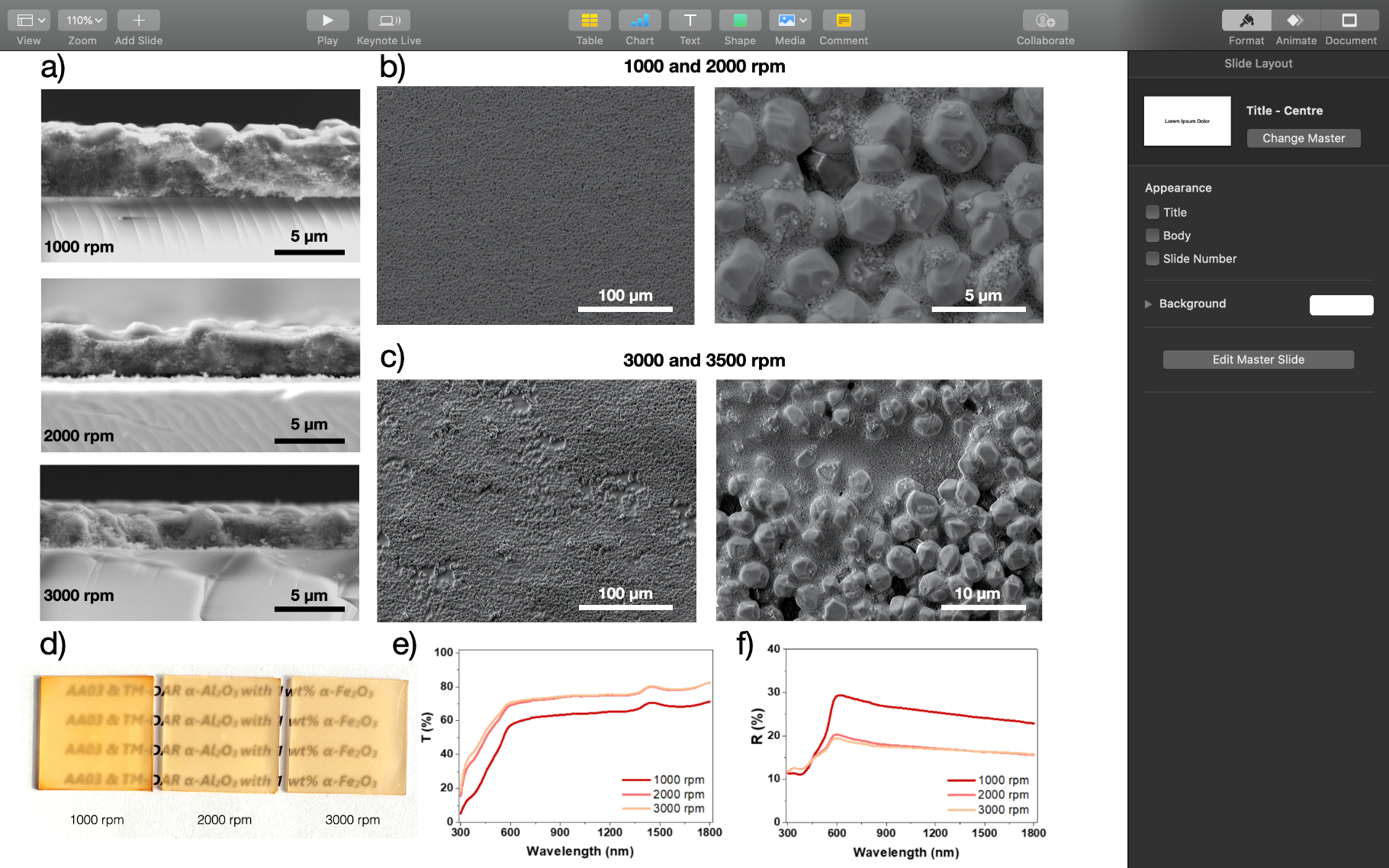


**Supplementary Fig.1**. Characterization of ceramic layers (α-Al_2_O_3_ bimodal mixture doped with 1wt% α-Fe_2_O_3_) fabricated by spin coating with different rotation speeds (from 1000, 2000, and 3000 rpm): a) cross-sectional SEM images, b) top-view SEM images revealing uniform coating with 1000 and 2000 rpm, c) top-view SEM images showing non-uniform coating at 3000 and above rpm used, d) Photographs of the spin coated layers, e) optical transmittance and f) reflectance spectra of the spin coated layers.

**
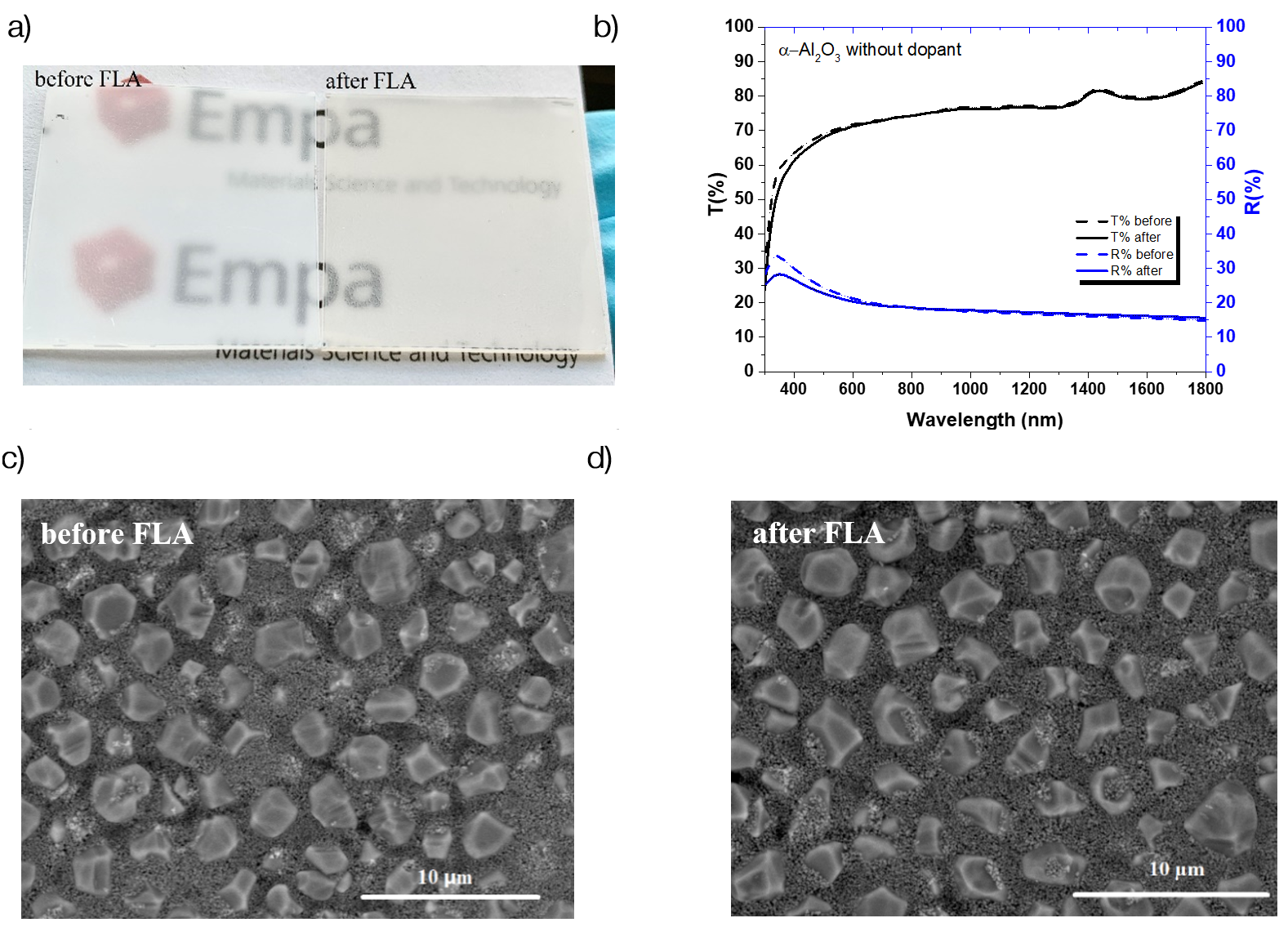
**

**Supplementary Fig.2**. Characterization of ceramic layers (α-Al_2_O_3_ bimodal mixture) without α-Fe_2_O_3_ dopant, before and after photonic sintering: top-view SEM images and optical transmittance and reflectance spectra. Photographs of the as-fabricated spin coated layer and layer after photonic sintering.

**a) b)**


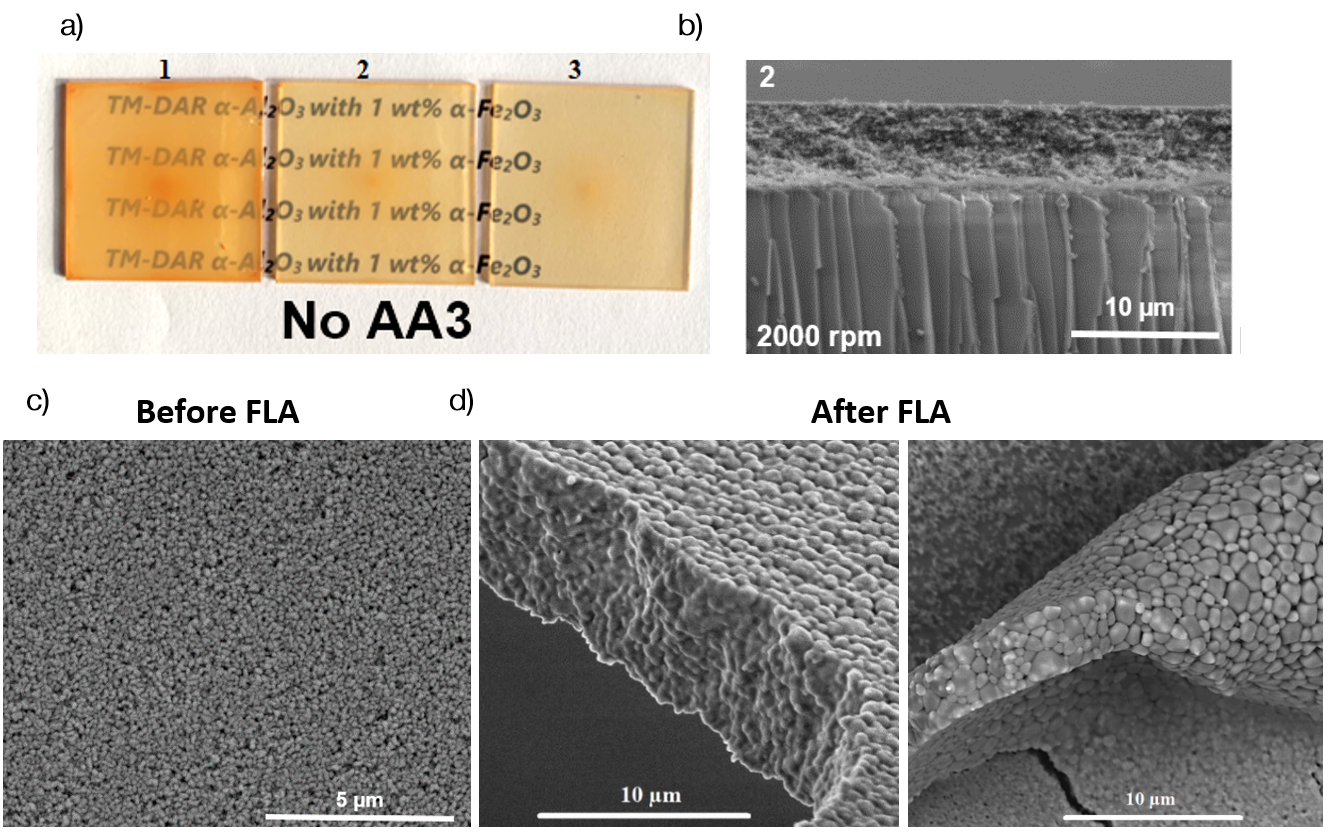


**Supplementary Fig.3**. Ceramic layers containing only nm-sized α-Al_2_O_3_ particles (no micrometer-sized AA3 particles) with α-Fe_2_O_3_ doping. a) Photographs of the ceramic layers spin coated with different rotation speeds (labeled 1, 2, 3), b) cross-sectional SEM image of the layer coating with 2000 rpm, c) top-view SEM images of the layers before and d) sintered after FLA (mostly delaminated or “wrapped” sintered films).

**
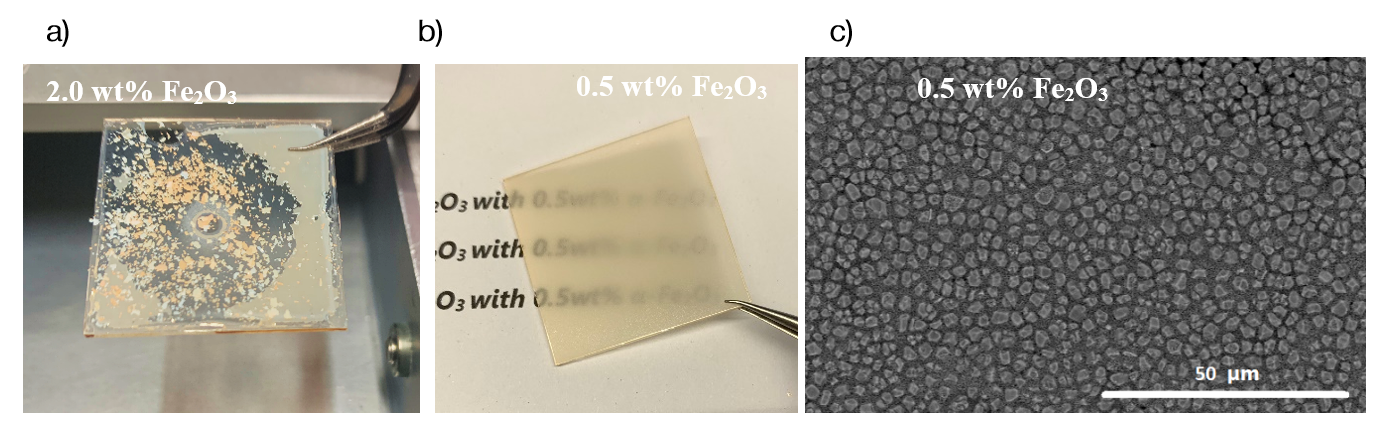
**

**Supplementary Fig.4.** Ceramic layers containing α-Al_2_O_3_ bimodal mixture with different amounts of the α-Fe_2_O_3_ dopant. Photographs of the FLA processed ceramic layers with a) 2.0 wt% and b) 0.5 wt% of Fe_2_O_3_ dopant. c) top-view SEM image of ceramic layer with 0.5 wt% after FLA. In the case of 2.0 wt% pulse conditions are too harsh and the samples are destroyed; for 0.5 wt% - the amount of dopant is too low and there is no effect on the layer after photonic sintering.


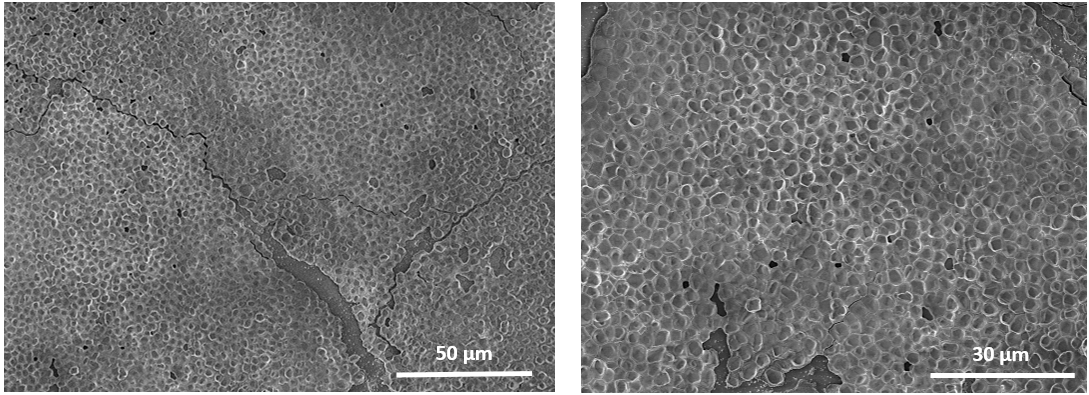


**Supplementary Fig.5.** Top-view SEM images of the large-area sintered layers with different magnifications.


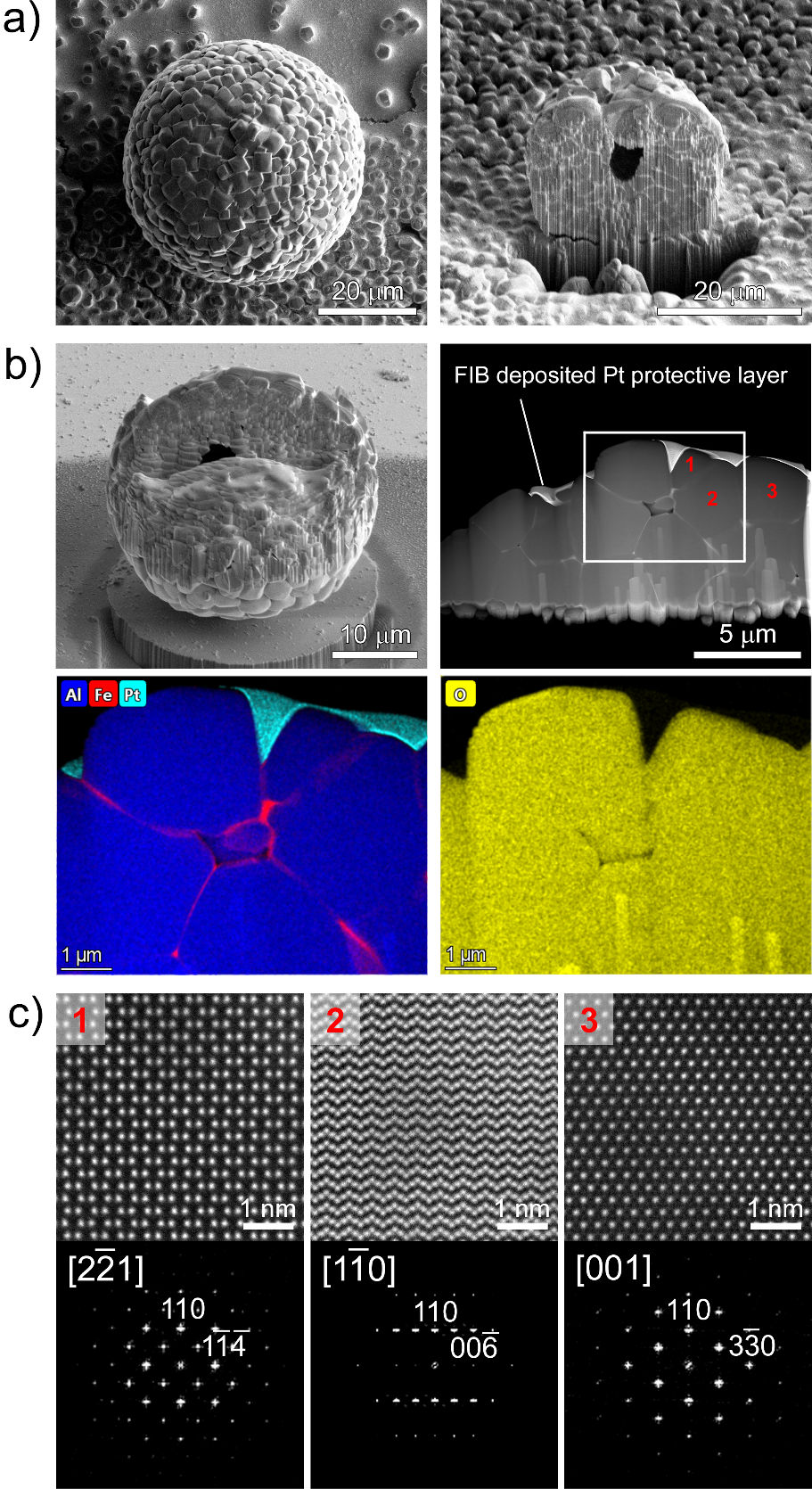


**Supplementary Fig. 6. Compositional and structural analysis of the α-Al_2_O_3_ spheres.** a, SEM images of sintered spheres (located at the edges of the sintered film). FIB cuts across the spheres reveal that they are hollow with wall thicknesses in the 3-10 µm range. b, SEM image showing the area where the FIB lamella was extracted and HAADF-STEM image of the FIB lamella. The EDX elemental maps (acquired from the white rectangle) of the FIB cross-section show the composition of the sintered sphere and the Fe localization at the grain boundaries. c, HAADF-STEM images acquired from the µm-sized α-Al_2_O_3_ particles indicated with 1-3 in panel b. The corresponding Fourier transforms of are all indexed using the α-phase of Al_2_O_3_.
